# Notes and News

**Published:** 1899-04-15

**Authors:** 


### Notes and News.

(Collected and Communicated.)

The Duchess of York will visit the West Norfolk and Lynn Hospital at Whitsuntide, when purses will be presented to her in aid of the Diamond Jubilee Children's Ward.

The grand bazaar in aid of St. Mary's Hospital is to be held at the New Hotel Great Central on June 6th and 7th.

A special meeting on behalf of the Buildings and Improvement Fund of the Royal Free Hospital is to be held on Tuesday, April 25th, at the Mansion House, at three p.m.

Extensive additions to the Preston and County of Lancaster Hospital are contemplated as a jubilee commemoration. The committee offered three prizes in competition for suitable plans. Those of Mr. Dixon, architect, of Preston, received the first prize of £50. The expenditure is estimated at slightly over £12,000.

A ball in aid of a seaside convalescent home for the London Hospital will be held at the Hotel Cecil on Thursday, April 20th. The ball promises to be a brilliant entertainment; it is under distinguished patronage, and tickets may be had from the hon. secretaries, Mrs. Spender, 29, Cheyne Walk, and Miss Gully, Speaker's House.

The foundation-stone of a new isolation hospital for Leicester has been laid. When completed, the hospital will consist of four pavilions of one storey for scarlet fever, with 26 beds in each; one pavilion for typhoid cases, with 28 beds; and two isolation pavilions, each with 28 beds, with the usual administrative buildings. Provision is made for later extension, the present expenditure being estimated at £54,000.

A grievance has been brought against Queen Charlotte's Hospital because a child of one of the inmates had been vaccinated with alleged evil consequences. Inquiry shows that the rules of the hospital provide that every child shall be vaccinated before leaving the institution unless the parents object. Care is taken that the only lymph used shall be the glycerinated calf lymph supplied by the Local Government Board.

An isolation hospital has been presented to the ratepayers of Gosport by Mr. and Mrs. T. N. Blake. The institution is built in three blocks, one for the administration, one for scarlet fever, and one for typhoid cases. Accommodation is provided for sixteen patients. A laundry, disinfecting apparatus, ambulance and van house, and mortuary are also provided. The hospital has been furnished through Mrs. Blake's generosity.

Lieutenant-General Sir John Stokes, K.C.B., issues an appeal for support for the Lady Strangford Hospital at Port Said. The contributions of shipowners and of persons interested in the welfare of our sailors, as well as the Board of Trade regulation allowance made for the sustenance of the patients, furnish a sufficient revenue for the maintenance of the hospital, but there is still a debt on the original building fund, and more buildings are required.

One of the pleasant announcements at the annual meeting of the Birmingham General Hospital was that in which Sir John Holder, through a letter to the chairman, made it known that he had acquired five properties adjoining the hospital site, and presented them to the institution. When the new hospital was erected it was obviously advisable to secure the land in question, but the owners demanded so large a price at the time that the project had to be abandoned. Through Sir John Holder's generosity the hospital is now in possession of property which will add to its present income, and render extension possible in the future.

The Council of the Medical Graduates' College and Polyclinic have decided that clinical consultations shall be held at the college, 22, Chenies Street, on Thursdays, April 20th and 27th, at four p.m., and that the consultations shall be continued during the month of May every Tuesday, Thursday, and Friday at the same hour. Patients must be recommended by a medical practitioner; but we believe that any medical practitioner, whether a member of the college or not, may send a case for consultation (of course, after previously communicating with the secretary as to the date), and that the result of the consultation will be communicated direct to the doctor by whom the patient is sent up.

The special meeting of governors of the Devon and Exeter Hospital dealt with a large number of questions of vital interest to all institutions of the kind. The Devon and Exeter Hospital has experienced a large amount of criticism recently, and a complete inquiry into its financial condition has taken place. At the meeting it was proposed to close one large ward to decrease the yearly deficit. Discussion brought out the fact that the capital of the hospital equalled upwards of £100,000, from which a yearly income of £3,000 was derived. In the face of this fact, a resolution in favour of resorting to other means of diminishing the adverse balance was passed, and it was also decided that a reinvestment of the capital should be considered. A proposal to open a paying ward, unfortunately, did not receive much attention, and another suggestion for the more satisfactory admission of patients met with no definite result. Nevertheless, the meeting showed that amongst the governing body a distinct advancement has been made in realising the measures which tend generally towards progress and judicious administration.

